# Diagnosis of Chronic Obstructive Pulmonary Disease and Regulatory Mechanism of miR-149-3p on Alveolar Inflammatory Factors and Expression of Surfactant Proteins A (SP-A) and D (SP-D) on Lung Surface Mediated by Wnt Pathway

**DOI:** 10.1155/2022/7205016

**Published:** 2022-04-12

**Authors:** Xiuli Zhang, Yaping Wang, Xiang He, Zerui Sun, Xuefeng Shi

**Affiliations:** ^1^Department of Respiratory Medicine, Qinghai Provincial People's Hospital, Xining, Qinghai 810007, China; ^2^Digestive Department, Wuxi Huishan District People's Hospital, Wuxi 214187, China

## Abstract

**Objective:**

To study the mechanism of chronic obstructive pulmonary disease (COPD) in diagnosing alveolar factors and analyze the effect of miR-149-3p on alveolar inflammatory factors and the expression of surfactant protein D (SP-D) and SP-A on the lung surface mediated by Wnt pathway.

**Methods:**

Patients with stable COPD were taken as the research subjects, and healthy volunteers as the control group. Cardiac color Doppler ultrasound was adopted to measure the ventricular structure of patients. The ultrasound simulation method was introduced in the ultrasound imaging. The ultrasound image was processed based on the intelligent ultrasound simulation algorithm. The changes in the structure of the left and right ventricles were analyzed and compared in the two groups. The expression changes of miR-149-3p, Wnt1, *β*-catenin, RhoA, and Wnt5a in lung tissues of mice in three groups were detected, as well as the content of tumor necrosis factor- (TNF-) *α*, IL-1*β*, interleukin (IL-6), nuclear factor kB (NF-kB), and other inflammatory factors in bronchoalveolar tissues of mice in three groups.

**Results:**

The position where the attenuation ratio was less than 0.92 in the experiment under the ultrasonic simulation algorithm had a gray value of 50. Compared with the control group, the right ventricular mass index of patients with stable COPD was statistically considerable (*P* < 0.05). In patients with stable COPD, the overall right ventricular longitudinal strain, right ventricular diastolic longitudinal strain rate (RV DLSR), right ventricular diastolic circumferential strain rate, and right ventricular longitudinal displacement were significantly impaired (*P* < 0.05). The content of miR-149-3p in the lung tissue of the model group was dramatically inferior to that of the control group and the interference group (*P* < 0.05). The contents of Wnt1, *β*-catenin, RhoA, and Wnt5a in the lung tissue of the model group were dramatically superior to those of the control group (*P* < 0.05). In addition, the expressions of TNF-*α*, IL-1*β*, IL-6, and NF-kB in the alveolar lavage fluid of the model group were statistically different from those of control group (*P* < 0.05). The expression levels of SP-D and surfactant protein A (SP-A) in the COPD group were also statistically different from those of control group (*P* < 0.05).

**Conclusion:**

miR-149-3p regulated the expression of Wnt1, *β*-catenin, RhoA, and Wnt5a, which also affected the signal transmission of the Wnt pathway, causing changes in the expression of alveolar inflammatory factors. Eventually, it affected the development of COPD.

## 1. Introduction

Chronic obstructive pulmonary disease (COPD) is a respiratory disease characterized by persistent and progressive airflow obstruction accompanied by significant systemic effects [1]. The chronic inflammatory responses to toxic gases or particles in airway and lung will promote the progressive development of airflow obstruction [2, 3]. The pathogenesis of COPD is relatively complex. Although there are a lot of studies, it has not been fully clarified, and the currently recognized pathogeneses include chronic inflammation, oxidation-antioxidant imbalance, and protease-antiprotease imbalance. Some scholars also believed that the pathogenesis of COPD is accomplished by the joint action of these three mechanisms. COPD is mainly caused by smoking, air pollution, occupational dust, harmful gas, and infection [4–6].

Severe obstructive ventilatory dysfunction will occur in the late stage of COPD. Because the reduction in forced vital capacity (FVC) may approach or exceed the reduction in forced expiratory volume (FEV1) in 1 s, it seems that the sensitive index used to determine the type of obstructive ventilatory dysfunction (the ratio of the forced expiratory volume in 1 s to the forced vital capacity, FEV1/FVC) is not as obvious as the early reduction [7]. FEV1 can only be utilized to judge the severity of ventilatory dysfunction, not the type of ventilatory dysfunction [8]. The pathogenesis of COPD is related to the activation, infiltration, and release of inflammatory factors by inflammatory cells to cause nonspecific airway inflammation. Related inflammatory factors include TNF-*α*, IL-1*β*, IL-6, and NF-kB [9]. The incidence and mortality of COPD are increasing year by year, posing a serious threat to people's life and health [10, 11].

At present, the mechanism of the initial pathological features and pathological changes of COPD caused by smoking and the repeated acute exacerbations of some patients after smoking cessation has not been clarified. Evaluation of COPD should include comprehensive pulmonary function, clinical manifestations, acute exacerbations, and comorbidities [12]. Ultrasound is a mature, safe, and noninvasive imaging diagnosis method that can dynamically observe specific lesions and can also guide operations such as needle biopsy, drug perfusion, freezing, and radiofrequency ablation. It shows a good accuracy rate for lung diseases [13–15].

miRNA is a kind of short-chain endogenous RNA that does not encode proteins and is 22 nucleotides in length, which is ubiquitous in eukaryotic organisms. It can be generated by DNA but cannot be translated into proteins. It has regulatory functions and can maintain certain stability in the extracellular environment of human body [16]. There are more than 1,000 types of miRNAs, mainly distributed in serum, urine, plasma, and sputum. Related studies found that miRNAs are related to related diseases of the respiratory system. For example, miR-21, miR-29a, and miR-221 play a role in non-small cell lung cancer [17, 18]. Some researchers found that the abnormality of miR-149-3p in peripheral blood is related to the prognosis of patients with non-small cell lung cancer. There are also documents showing that the abnormality of miR-149-3p may affect the prognosis of patients through DNA topoisomerase 1. Studies pointed out that miR-149 plays an important role in inhibiting inflammation [19].

Pulmonary surfactant protein A (SP-A) and lung surfactant protein D (SP-D) belong to pulmonary surfactant protein (SP). SP-A and SP-D can participate in the elimination of inflammatory factors in the lungs and can regulate the function of immune cells, thereby regulating innateness, acquired host immune defense, and acute and chronic inflammation. In clinical practice, some indicators are observed, and the active proteins and inflammatory factors in the lungs are monitored [20].

Abnormal miRNA expression in COPD patients can explain the mechanism of COPD occurrence and development from different perspectives. In this way, the regulation content of related molecular levels in respiratory diseases is enriched, and it provides research directions for the diagnosis and treatment of lung diseases in the clinic. In this study, the regulatory mechanism of miR-149-3p on alveolar inflammatory factors and the expression of SP-A and SP-D on the lung surface mediated by the Wnt pathway was studied. In-depth discussion of the related content of miR-149-3p in the field of COPD was made, hoping to provide a theoretical basis for the next step on the functional mechanism of differential genes and target genes in COPD disease.

## 2. Research Content and Methods

### 2.1. miRNA Overview

**Figure 1:**
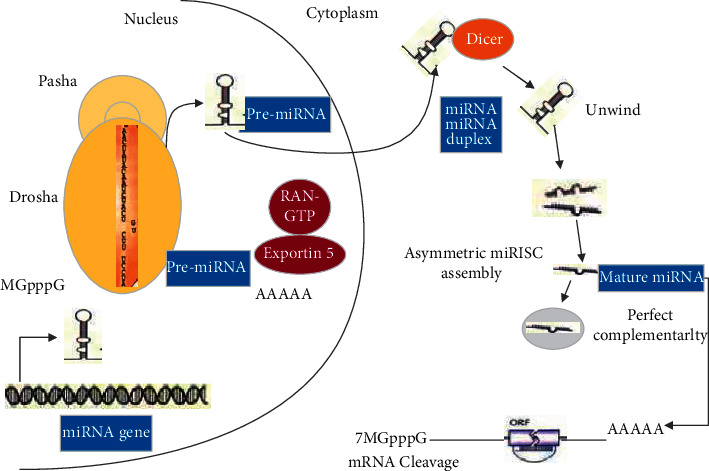
Schematic diagram of the main structure of miRNA.

miRNA is a type of noncoding single-stranded RNA molecule with a length of about 20–24 nucleotides encoded by endogenous genes. It is mainly involved in the regulation of posttranscriptional gene expression in animals and plants. So far, 18,645 miRNAs have been found in animals, plants, and viruses. Most miRNA genes exist in the genome in the form of single copy, multiple copies, or gene clusters. According to related speculations, noncoding small RNAs are involved in gene expression, but the mechanism is different from siRNA (double-stranded) mediated mRNA degradation. The first confirmed miRNA was found in *C. elegans* for the first time, and then the research team including humans, fruit flies, plants, and other biological species identified hundreds of miRNAs (Figure 1).

**Figure 2:**
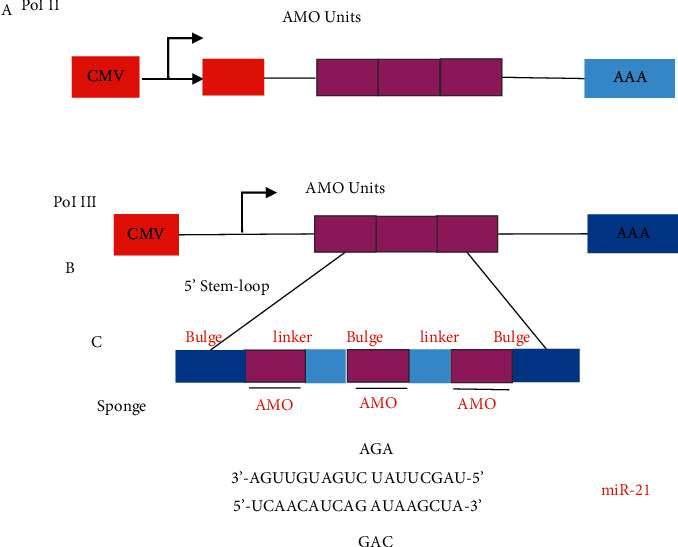
Schematic diagram of miRNA sponge principle.

Figure 2 shows a schematic diagram of the principle of miRNA sponge. Upregulation of miRNA is adopted to identify the gain-of-function phenotype, and suppression or downregulation is adopted to study the loss-of-function phenotype. The combination of upregulation and downregulation is utilized to identify genes regulated by specific miRNAs and cell processes involved in specific miRNAs.

### 2.2. Clinical Data of Research Objects

A total of 50 patients diagnosed with COPD admitted to our hospital were selected. Patients with stable COPD were taken as the research objects, and 50 healthy volunteers with matching gender, age, and body mass index (BMI) were taken as the control group. The research experiment had been approved by the hospital ethics committee. Diagnostic criteria are as follows: (i) patients had complete pulmonary function tests; (ii) patients had COPD symptoms, chronic cough, expectoration, and dyspnea; (iii) irreversible airflow obstruction was confirmed in patients with FEV1/FVC less than 70% after bronchodilator administration; (iv) patients had perfect chest X-ray examination, which excluded lung cancer, pulmonary fibrosis, bronchial asthma, and other respiratory difficulties; (v) the patient had stable symptoms such as shortness of breath, expectoration, and cough, or mild symptoms. Inclusion criteria are as follows: (i) patients who had no other major diseases of the organ system; (ii) the patient who met the clinical COPD diagnostic criteria, and there was no cough, sputum, and asthma symptoms aggravation for more than one month; (iii) the patient who had no other respiratory diseases; and (iv) the patient who received no other treatment except bronchodilator/glucocorticoid inhalation more than one month before blood collection.

Exclusion criteria are as follows: (i) patients who had incomplete clinical data; (ii) researchers who did not voluntarily participate in this study; (iii) patients who had complications in heart, liver, or kidney; and (iv) patients who had mental disease.

Inclusion criteria of volunteers are as follows: (i) respiratory system function of all volunteers met the corresponding health standards; (ii) the average age difference between the volunteers and the enrolled COPD group was no more than five years; (iii) all volunteers signed the informed consent; and (iv) the study had been approved by the hospital ethics committee.

Exclusion criteria for healthy volunteers are as follows: those who were unwilling to participate in this study.

### 2.3. Ultrasound Simulation Algorithm

The simulation of ultrasound image is to simulate the reflection of ultrasound based on the reflectivity of ultrasound. The reflection characteristic of ultrasound means that when the sound wave propagates to different tissue contact surfaces, a part of the sound wave will be reflected. This phenomenon occurs due to the acoustic impedance of the body. The acoustic reactance equation was expressed as follows:

(1) $$\begin{array}{c} Q=\rho V. \end{array}$$

*V* represented the propagation speed of sound waves, *Q* represented the acoustic impedance, and *ρ* represented the density of the tissue. If there was a specularly reflected sound wave, the *Q*_1_ and *Q*_2_ could be adopted to represent the impedance of two adjacent tissues, respectively, and then the reflection intensity of the sound wave was expressed as

(2) $$\begin{array}{c} K=\frac{{\left(Q_{2}-Q_{1}\right)}^{2}}{{\left(Q_{2}+Q_{1}\right)}^{2}}. \end{array}$$

The ultrasonic probe used diffuse reflection when the echo was reflected, and the intensity ratio of the received sound wave reflection would be affected by the angle of incidence. The diffuse reflection intensity ratio could be calculated with the following equation:

(3) $$\begin{array}{c} R={\left(\cos\;\theta \right)}^{n}{\left(\frac{Q_{2}-Q_{1}}{Q_{2}+Q_{1}}\right)}^{2}. \end{array}$$

In the equation above, *n* indicated that the diffuse reflectance ratio of the interface of different tissues was different, which was related to the degree of simulation of image details. The X-ray attenuation value *δ* was defined to replace *Q* in the calculation process; then the below equation could be obtained:

(4) $$\begin{array}{c} R={\left({\overset{⟶}{d}}^{T}\frac{\nabla \delta \left(\overset{⟶}{x}\right)}{\left|\nabla \delta \left(\overset{⟶}{x}\right)\right|}\right)}^{n}\left(\frac{\nabla \delta \left(\overset{⟶}{x}\right)}{2\max\left(\delta \left(\overset{⟶}{x}\right)\right)}\right), \end{array}$$

where $\overset{⟶}{d}$ represented the unit vector in the direction of the sound wave, and $\delta \left(\overset{⟶}{x}\right)$ referred to the attenuation value. If *n* = 1, the equation for calculating the transmittance was as follows:

(5) $$\begin{array}{c} t=1-{\left(\frac{\left|\nabla \delta \left(\overset{⟶}{x}\right)\right|}{2\max\left(\delta \left(\overset{⟶}{x}\right)\right)}\right)}^{2}. \end{array}$$

The transmission intensity was attenuated during the transmission of the sound wave. Equation (6) could be adopted to calculate the attenuation ratio of the ultrasonic intensity when the sound wave reached each position.

(6) $$\begin{array}{c} I_{X}=I_{0}\exp\left(-\int_{0}^{\lambda x}{\left(\frac{\left|\nabla \delta \left(\overset{⟶}{x}+\lambda \overset{⟶}{d}\right)\right|}{2\max\left(\delta \left(\overset{⟶}{x}+\lambda \overset{⟶}{d}\right)\right)}\right)}^{2}\lambda \;d\right). \end{array}$$

The attenuation ratio of each position could be calculated using differences, the calculation amount was relatively large, and the simulation speed was low. The use of the simulation algorithm can shorten the calculation time, increase the simulation speed, and have higher accuracy in the registration process.

### 2.4. Treatment Methods

All the included research subjects were tested by color Doppler ultrasound and Holter. Forced expiratory volume in 1 s/forced vital capacity (FEV1/FVC) in one second of patients with stable COPD were collected within 48 hours. Then, the changes of left and right ventricular structure and function parameters and right ventricular myocardial deformation parameters were analyzed in the two groups. Thirty SPF male healthy mice were randomly divided into Con group (blank control), model (COPD) group, and interference group (COPD model + anti-miR-149-3p), with ten mice each. Mice were sacrificed, and lung tissues were taken. Then, quantitative polymerase chain reaction (qPCR) was performed to detect the expression changes of miR-149-3p and Wnt1, *β*-catenin, RhoA, and Wnt5a in lung tissues of three groups of mice. Enzyme-linked immunosorbent assay (ELISA) was adopted to detect the levels of inflammatory factors such as tumor necrosis factor- (TNF-) *α*, IL-1*β*, interleukin (IL-6), nuclear factor kB (NF-kB), SP-A, and SP-D in bronchoalveolar lavage fluid.

Experimental environment was defined as follows. The image under study was 521 × 512 × 344 pixels, the layer spacing was 0.6 mm, and the distance between pixels was 0.59. The same expert was invited to judge the ultrasound image, save the image in dicom format, use a computer to obtain the attenuation value, and adopt a medical image segmentation and registration toolkit and C++ language.

### 2.5. Enzyme-Linked Immunosorbent Assay (ELISA)

- Solution preparation: the required lath was taken from the aluminum foil bag after being balanced at room temperature for half an hour, the remaining part was put back in the refrigerator at 4°C, and the wash solution was diluted with double distilled water at a concentration ratio of (1 : 25).
- Standard substance (Table 1): 1.0 mL standard substance and sample dilution were added to the freeze-dried standard substance and left for ten minutes. It was turned upside down several times and then mixed with standard substance. According to the dilution multiple dilutions, the blank hole was the sample diluent.
- Enzyme binding working solution: 100 *μ*L was added per well, the required amount was calculated, and 100 *μ*L more was prepared. The concentration of horseradish peroxidase (HRP) binder was diluted at 1 : 100 to the concentration required in the test using enzyme binder diluent 15 minutes prior to the test.
- Antibody working fluid biotinylation: 100 *μ*L was added per well, the required amount was calculated, and 100 *μ*L more was prepared. Biotinylated antibody dilution was utilized to dilute the concentration of biotinylated antibody 1 : 100 to the concentration required for the experiment 15 minutes prior to the experiment.
- The plate was soaked in the washing solution for 1-2 min, and then it was washed with water and put into the oven to dry with clean water.
- Sample loading test: the standard well, blank well, and test well were added with sample, and 100 *μ*L of the corresponding standard or dilution was added to each well. (It was important to note that the sample was added to the bottom of the plate, which was gently shaken and mixed to avoid bubbles, and the wall of the hole cannot be touched.) The nozzle sucked the liquid from the well, and 100 *μ*L biotinylated antibody working fluid was added to the well. The plate was covered with membrane and placed at 37°C for 1 hour. The nozzle was utilized to absorb the liquid in the hole. After the enzyme plate was clean, the blotting paper was utilized to blot the liquid in the hole. 100 *μ*L of enzyme-bound liquid was added to each empty plate, and the plate was covered with a membrane, as described above.

### 2.6. Animal Model Construction

Thirty healthy adult SPF male mice with an average body weight of 258 ± 28 g were selected as subjects.

All the mice were fed normally for one week, drinking water freely, feeding temperature was 24°C–26°C, and humidity was 40%–60%. Thirty mice were divided into control group (no treatment), model group (COPD model), and interference group (COPD model + anti-miR-149-3p) by random number table method, with ten mice in each group. After one week of adaptive feeding, the animal model of COPD mice was established by intranasal infusion of lipopolysaccharide (LPS, Sigma) plus smoking cigarette. On days 1 and 29, the intranasal infusion of LPS (30 *μ*g/6 *μ*L) was made, and on days 2–30 (except for days 29), smoking was used. Mice were placed in a homemade glass fumigator, with 10 at a time, for 30 minutes, five days a week, lasting for four weeks.

In the feeding of mice, the general observation showed that the blank control group mice did not die, with hair luster, uniform breathing, and normal activities. Mice in model group had dull hair; some of them had hair removal and were restless, gathering, tired and curled up, and sweating out. In addition, there was abdominal swelling, shortness of breath, nodding movement, and even open mouth breathing.

Lung morphology was detected as follows. After abdominal anesthesia, the mice were exposed to thoracotomy, and the left lung lobe was fixed with 10% formaldehyde solution for 24 hours. Conventional dehydrated paraffin embedding was implemented, so did section HE staining and observation under light microscope. In the blank control group, the alveolar structure was clear, the alveolar size was uniform, the airway mucosal epithelial structure was intact, and the cilia were orderly arranged. In model group, some alveolar walls collapsed, and alveoli expanded irregularly and fused with each other to form bullae. Different degrees of inflammatory cell infiltration were seen in the lung interstitium in the two groups. The epithelium of bronchial mucosa fell off; mucosal folds increased, protruding into lumen, with cilia adhered and lumen narrowed, which were consistent with the pathological changes of COPD.

The mice were sacrificed 24 hours after the last smoking and were anesthetized with 1% pentobarbital (0.05 ml/10g). Bronchoalveolar lavage fluid (BALF) was procured as described previously. BALF supernatants were kept at –80°C for future usel. The levels of TNF-*α*, IL-1*β*, IL-6, NF-kB, SP-A, and SP-D in bronchoalveolar lavage fluid were measured according to the ELISA kit instructions.

### 2.7. Animal Grouping and Handling

To simulate the stimulation of smoking on the lungs and other respiratory tract tissues, long-term mice with cigarette smoke were used to construct a model. According to different treatment methods, the mice were randomly divided into three groups, and each group was with ten mice.

  Group A (control group, *n* = 10): the healthy mice without any treatment.
  Group B (COPD group, *n* = 10): based on the treatments of the observation group, cigarettes were lighted in the observation room, and 20 cigarettes were lit up within 30 minutes, and 5 mL·kg^−1^·d^−1^ normal saline was injected through caudal vein after successful modeling.
  Group C (COPD+anti-miR-149-3p group, *n* = 10): COPD model + anti-miR-149-3p. 0.1 mL lentiviral vector overexpressing anti-miR-149-3p was injected through tail vein after successful modeling.

### 2.8. PCR Amplification Theory and Lung Tissue Detection

Quantitative determination is used in the dynamic observation of the results of antiviral treatment of patients with known viral infections and clinical research of antiviral drugs. It is also used in the research of gene expression such as the quantitative determination of specific mRNA. Quantitative PCR can obtain very accurate results. Even the differences between tubes can be eliminated, but the differences between samples cannot be eliminated. The accuracy of quantitative determination is much more important than the lower limit of determination. Quantitative determination is carried out during the exponential phase of amplification. Therefore, it is more important that every parameter involved in the entire amplification process is idealized to control the entire amplification.

PCR amplification is exponential amplification, and the amount of product after each amplification cycle is expressed as follows:

(7) $$\begin{array}{c} Y_{n}=\;Y_{n-1}\cdot \left(1+E\right)=Y_{n-2}\cdot {\left(1+E\right)}^{2}=\cdots =X\cdot {\left(1+E\right)}^{n},\;0\leq E\leq 1, \end{array}$$

where *E* represents the amplification efficiency, *Y*_*n*_ represents the number of PCR product molecules after *n* cycles, and *Y*_*n*−1_ is the number of PCR product molecules after *n*−1 cycle.

The equation holds only for a limited number of amplification cycles, generally 20–30. Beyond this number of cycles, the amplification process decreases from exponential amplification to a stable amplification rate and finally reaches a plateau where no amplification occurs.

In this subsection, we described the PCR method.

### 2.9. Statistical Analysis

SPSS 21.0 was used for statistical processing of experimental data. Normality test and variance test were carried out for measurement data. The data subjected to normal distribution and homogeneity of variance were represented by mean ± standard deviation ($\bar{x}\pm s$). The chi-square test and *T* test were utilized to compare the count data between groups. The count data was expressed as percentage (*N*, %). The change of each index was analyzed by repeated measurement design ANOVA. The receiver operating characteristic (ROC) curve was utilized to analyze the sensitivity and specificity of sensitive indicators. The data were tested for normality and spherical symmetry, and the test level was set at 0.05. *P* < 0.05 was statistically considerable. Intergroup and intragroup consistency analysis of the parameters involved in the study was performed using ICC test and statistical analysis was performed within the 95% confidence interval.

## 3. Results

### 3.1. Ultrasound Image

**Figure 3:**
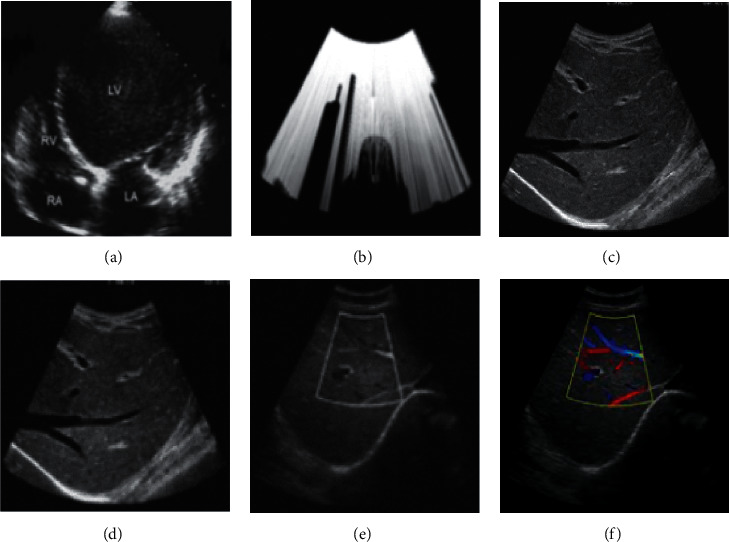
Ultrasound image and propagation attenuation image.

Image A in Figure 3 is the original ultrasound picture, and image B is the attenuation image of the ultrasonic sound wave in the propagation process. There were some white spots in image B, which may be because the image has not been processed, and the white dot indicates that the definition of the image is low, and the image magnification will disappear, but the image quality is blurred. Images C and F were the images after processing by the algorithms, and they were relatively clear. Images D and E showed the images before processing using the intelligent algorithms, and the images contained graininess. The image comparison showed that the image clarity was significantly improved after processing by the intelligent algorithms. The yellow boxes in image F indicate the location of the lesion.

Table 2 results are shown in 3.2.

### 3.2. Comparison of General Information and Lung Function between Groups

There was no significant difference in age distribution and gender composition among the three groups of study subjects (*P* > 0.05). The smoking index in COPD group was higher than that of control group. However, FEV1% predicted value, and FEV1/FVC in COPD group was significantly lower than control group (Table 2).

### 3.3. Comparison of Right Ventricular Mass Index

The independent sample *T* test was utilized to compare the right ventricular function indexes of the healthy control group and the stable COPD group. The results are shown in Table 3. Right ventricular Global longitudinal strain(GLS), right ventricular diastolic longitudinal strain rate (RV DLSR), global diastolic circumferential strain rate (DLSR), and right ventricular longitudinal displacement (LD) were significantly impaired in COPD patients (*P* < 0.05).

### 3.4. Specificity and Sensitivity Assessment

**Figure 4:**
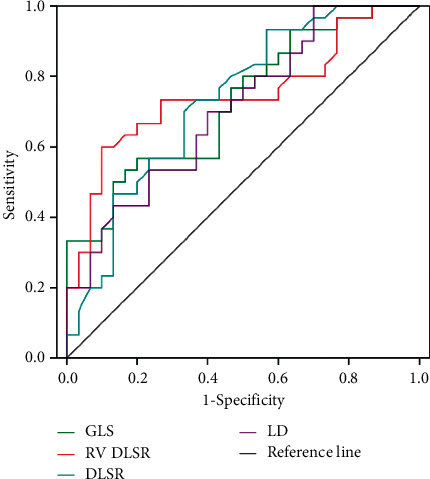
ROC curve.

The sensitivity and specificity of LD, GLS, RV DLSR, and DLSR to the psychopathological changes of the right ventricle were explored in patients with stable COPD. The results are shown in Table 4, and the ROC curve is shown in Figure 4. The areas under the curve of LD, GLS, RV DLSR, and DLSR were all above 0.7.

### 3.5. Lung Tissue mRNA Expression

**Figure 5:**
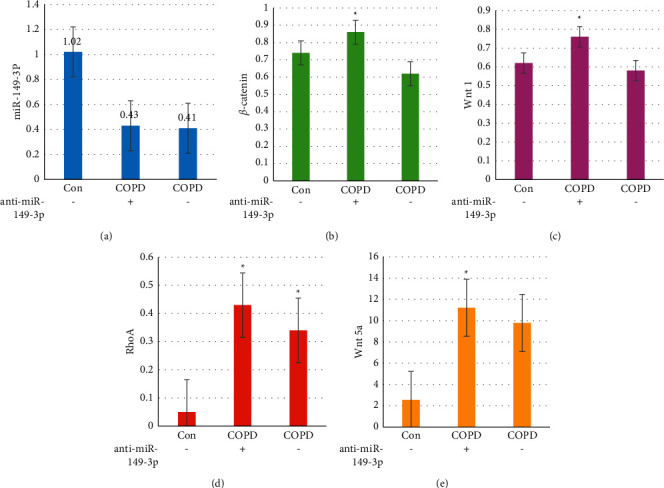
Results of relative expression of protein in lung tissue of rats in each group. Note: ^*∗*^ indicated that the difference was considerable, *P* < 0.05.

Mice lung tissues were prepared, and qPCR was implemented to detect the expression of miR-149-3p, Wnt1, *β*-catenin, RhoA, and Wnt5a in the lung tissues of the three groups of mice, as shown in Figure 5. The relative expressions of miR-149-3p, Wnt1, *β*-catenin, RhoA, and Wnt5a in the lung tissue of the model group were dramatically superior to those in the Con group and COPD+anti-miR-149-3p group (*P* < 0.05).

### 3.6. SP-A and SP-D Expression Level

The measurement results of SP-A and SP-D in the Con group and the COPD group are shown in Table 5. The expression levels of SP-D and SP-A in COPD patients was higher than that in healthy objects (*P* < 0.05).

### 3.7. Hematoxylin and Eosin (HE) Staining

**Figure 6:**
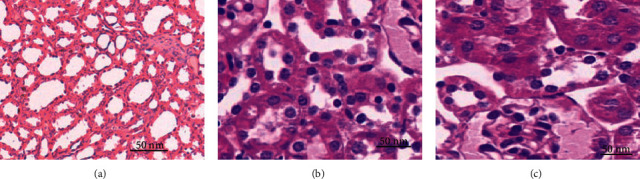
HE staining results of cell apoptosis in lung tissues of mice in each group. (a) Control group; (b) COPD+anti-miR-149-3p group; (c) COPD group.

HE staining was performed to detect the apoptosis of lung tissue in each group.  Figure 6 shows the optical microscope observation of HE staining (×400). Five visual fields were randomly selected in each slice for observation.  Figure 6 shows a statistical chart of apoptotic cells. The number of tissue cell apoptosis in Figures 6(b) and 6(c) was dramatically superior to that in Figure 6(a), indicating that miR-149-3p had a certain inhibitory effect on cell apoptosis. The content of miR-149-3p in lung tissue of COPD group was dramatically inferior to that of Con group and COPD+anti-miR-149-3p group (*P* < 0.05).

### 3.8. Comparison of Three Groups of Inflammatory Factors

**Figure 7:**
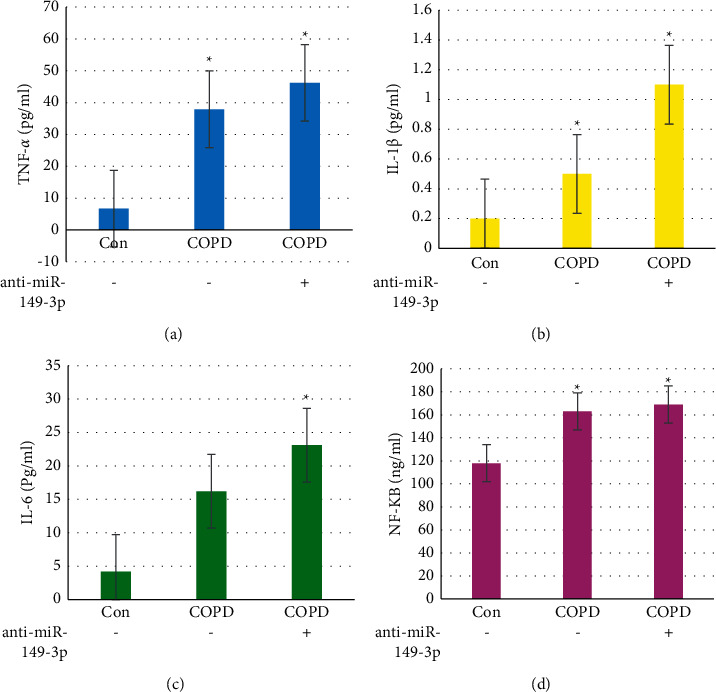
Results of alveolar inflammatory factors in each group of mice. Note: ^*∗*^ indicated that the difference was considerable (*P* < 0.05).

Figure 7(a) shows the result graph of TNF-*α* expression, and Figure 7(b) shows the result graph of IL-1*β* expression.  Figure 7(c) shows the IL-6 expression level result graph, and Figure 7(d) shows the NF-kB expression level result graph. The expression of TNF-a, IL-1*β*, IL-6, and NF-KB in COPD and COPD+anti-miR-149-3p group were higher than control group, and TNF-a, IL-1*β*, IL-1, and NF-KB level in COPD+anti-miR-149-3p group was higher than COPD group. Compared with the control group, the expressions of TNF-*α*, IL-1*β*, IL-6, and NF-kB in the alveolar lavage fluid of the model group were statistically poor (*P* < 0.05).

As shown in Table 6, SP-A and SP-D levels were significantly higher in BALF of COPD and COPD+anti-miR-149-3p group than in control group (*p* < 0.05).

## 4. Discussion

The development of COPD is related to the release of many inflammatory factors and inflammatory cells. Related inflammatory factors include TNF-*α*, IL-1*β*, IL-6, NF-kB, and interleukins [21, 22]. TNF-*α* is the most important mediator of airway inflammation. If T lymphocytes and mononuclear macrophages are produced, the inflammatory effects of IL-8 and neutrophils are activated [23]. IL-6 and IL-8 are directly involved in the inflammatory injury of normal airway mucosal epithelium, which induce neutrophil infiltration and promote the secretion of C-reactive protein in the liver [24, 25]. Studies suggested that normal people's inflammatory factors were dramatically inferior to those of COPD patients, and they had a certain relationship with the quality of life of patients [26, 27]. The increase in pulmonary artery pressure in patients with COPD, lung hyperinflation, vascular endothelial abnormalities, and systemic inflammation all indirectly or directly affected the increase in right ventricular load. The ROC curve in this study showed that the areas under the curve of the right heart pathophysiological changes were all above 0.7. Easter et al. [28] analyzed the left ventricular structure. Myocardial interstitial fibrosis varied with the phenotype of the left ventricle of hypertension. Before the occurrence of eccentric hypertrophy of the left ventricle, the left ventricular ejection fraction was higher than normal. Whether the right ventricular configuration of COPD patients is the same as that of the left ventricle is still uncertain.

Wnt protein is a group of secreted glycoproteins rich in cysteine. Wnt-1 is a signal molecule in the Wnts/*β*-catenin pathway. Wnts and their receptors have very complex expression patterns, and they also play a very important regulatory role. *β*-Catenin is an important part of the Wnt signaling pathway. It is at the center of the entire pathway and plays a decisive role in the richness of neuron precursor cells. In this study, the expression levels of TNF-*α*, L-1*β*, IL-6, and NF-kB in serum of three groups of mice of Con, COPD, and COPD+anti-miR-149-3p were measured. The expressions of TNF-*α*, L-1*β*, IL-6, and NF-kB in the serum of mice in the COPD group were statistically higher than those of Con group (*P* < 0.05). Overexpression of Wnts or ectopic expression of activated *β*-catenin can cause the volume of nerve block to increase. Wnt5a can be used as a signal for cell growth and differentiation in two forms of autocrine or paracrine. Wnt5a can activate the classic Wnts/*β*-catenin pathway and the noncanonical Wnts/Ca^2+^ pathway. The interaction between the two can play a very important role in cell differentiation and maturation [29].

Bowin et al. [30] analyzed miRNAs and showed that the expression of miR-149-3p in nonsmoking non-COPD was higher than smoking non-COPD, smoking with acute exacerbation COPD, and smoking with stable COPD. Cigarette smoke stimulation downregulated the expression of miR-149-3p in THP-1 cells and upregulated the levels of TLR-4 and NF-kB. Transfection of mir-R-149-3p inhibitor in TLR-4 cells also increased the expression of its target genes. Overexpression of miR-149-3p inhibited the NF-kB signaling pathway, reduced the secretion of L-1*β* and TNF-*α*, and reduced the response of inflammatory factors. In this study, the results showed that the expression levels of TNF-*α*, IL-1*β*, IL-6, and NF-kB in the serum of the COPD+anti-miR-149-5p group were statistically higher than those in the control group (*P* < 0.05).

With artificial intelligence algorithms, humans can use computers to simulate intelligent control functions. Computers are used to imitate human brain thinking, recognition, reasoning, and problem-solving thinking activities, intelligently processing ultrasound images. Appropriate algorithms can provide more accurate images with more reasonable quality [31]. The innovative artificial intelligence architecture proposed by Li et al. [32] can automatically integrate ultrasound and shear wave elastic information, and artificial intelligence can provide more significant diagnostic performance improvements for radiology departments and above. Al-Kadi et al. [33], based on nonadaptive three-dimensional multiresolution feature description, adopted the ultrasound radio frequency data set to predict the intensity change of image texture, which can promote disease treatment. Tian et al. [34] believed that, in the phacoemulsification surgery, ultrasound intelligently recognized various information in the image diagnosis system and the intelligent decision maker in ultrasound can identify the level of disease in complex videos. This research used intelligent ultrasound algorithms to process images, showing good results, and image clarity and marginalization had been well improved.

## 5. Conclusion

In this study, the addition of simulated short hair in the ultrasound imaging process increases the reliability of the calculation and reduced the amount of calculation. The diagnosis of patients with stable COPD and the influence of miR-149-3p on inflammatory factors. The right ventricular myocardial deformation parameters of COPD group were were significantly impaired (*P* < 0.05).

miR-149-3p affects the Wnt pathway signal transmission by regulating the expression of Wnt1, *β*-catenin, RhoA, and Wnt5a. Inhibition of miR-149-3p increased the secretion of TNF-a, IL-1*β*, IL-6, NF-KB, SP-A, as well as SP-D, and promote inflammatory response in lung tissue by Wnt signaling pathway. In short, this work provides a reference for the diagnosis and treatment of COPD. The results suggested that the Wnt pathway in vivo is a complex regulatory network. Various dialogues exist between each signal and inflammatory factors, which synergistically participate in the regulation of miR-149-3p on inflammatory factors. However, clinical trials are needed to confirm this in future studies.

## Figures and Tables

**Table 1:** Standard content of detection factors.

Detect factors	Standard substance							
	1	2	3	4	5	6	7	8

TNF-*α*	5000	2500	1250	625	312.5	156.25	78.125	0
IL-1*β*	2000	1000	500	250	125	62.5	31.25	0
IL-6	2500	1250	625	312	156	78	78	0
NF-kB	5000	2500	1000	500	200	100	50	0

**Table 2:** Comparison of general information and lung function between groups.

Item	CON	COPD	Statistics	*P*

Age (years)	66.26 ± 8.51	65.84 ± 7.00	0.816	0.444
Gender (case/%)				
Male	33 (66%)	34 (68%)	0.434	0.805
Female	17 (34%)	16 (32%)		
Smoking index [*M* (*Q*_1_, *Q*_3_)]	265 (120, 340)	420^a^ (320, 585)	28.612	<0.001
Number of acute exacerbations in the past year [times, *M* (*Q*_1_, *Q*_3_)]	—	1.0 (1.0, 2.0)	−3.802	<0.001
mMRC score [score, *M* (*Q*_1_, *Q*_3_)]	—	1.0 (1.0, 2.0)	−6.259	<0.001
FEV1% predicted value	80.69 ± 7.62	54.21 ± 6.76	277.513	<0.001
FEV1/FVC (%)	83.44 ± 5.96	51.56 ± 6.29^a^	373.072^*∗*^	<0.001

^a^ *P* < 0.05, compared with the Con group; ^*∗*^indicates Welch analysis of variance.

**Table 3:** Two groups of right ventricular indexes.

Item	Control group	COPD group	t	*P*
GLS	−22.52 ± 4.16	−18.91 ± 4.63	3.584	0.001
RV DLSR	1.91 ± 0.46	1.39 ± 0.59	3.866	0.000
DLSR	1.86 ± 0.54	1.44 ± 0.47	3.259	0.002
LD	7.83 ± 2.43	5.98 ± 2.19	3.096	0.003

Note:  ^*∗*^*P* < 0.05,  ^*∗∗*^*P* < 0.01.

**Table 4:** Specificity and sensitivity assessment.

Item	AUC	SE	95% CI	Cut-off	Se (%)	Sp (%)	*P*
GLS	0.726	0.065	0.619, 0.875	−19.465	57	80	0.001
RV DLSR	0.747	0.065	0.605, 0.859	1.435	60	90	0.002
DLSR	0.732	0.064	0.623, 0.875	1.705	70	67	0.001
LD	0.707	0.066	0.578, 0.837	7.110	70	60	0.006

Note: CI: 95% confidence interval; Se: sensitivity; Sp: specificity.

**Table 5:** Two groups of SP-A and SP-D measurement results.

Serial number	SP-D (ng/mL)		SP-A (ng/mL)	
	Con	COPD	Con	COPD
1	8.558468	28.2358871	10.66308	18.30645
2	9.707661	17.71169355	10.40323	13.41398
3	21.13911	69.81854839	10.98566	66.33513
4	10.53427	27.0766129	23.98746	27.05197
5	22.06653	13.61895161	12.06989	11.36201
6	11.07863	9.606854839	17.22222	15.34213
7	11.5625	18.40501792	15.83984	12.36328
8	8.183594	38.58359375	1.601563	38.28125
9	0.214844	35.3125	1.699219	5.46875
10	0.234375	5.2734375	14.16016	49.02344
11	11.44531	51.953125	11.30859	11.89453
12	6.425781	31.651135	7.673081	17.10645
13	9.156457	38.13558708	9.433126	19.91498
14	6.502361	47.51169335	5.915761	69.13523
15	19.23321	61.41154829	21.38143	37.15297
16	11.33124	29.9761129	12.31589	31.16211
17	20.07523	18.61716161	16.66722	14.14216
18	13.17813	29.00685484	14.77684	32.16358
19	8.0645	38.55050179	5.510567	42.18127
20	12.18309	28.48369315	7.641219	35.56814
21	1.212254	55.1167	10.24116	59.42544
22	3.274585	35.2754771	6.104774	41.69353
23	14.43545	59.157179	13.31659	54.24117
24	4.445181	37.154137	6.773082	33.56458
25	8.930212	29.174136	9.149126	32.48224
26	18.55847	38.5158851	20.66308	48.80245
27	6.717564	19.92364355	9.503526	23.51318
28	20.63613	65.41153839	16.18466	65.12513
29	20.43428	47.0911129	23.18145	44.35127
30	12.06323	23.11495168	12.76181	31.56211
31	21.02842	29.50285184	17.34567	35.39116
32	12.1635	28.10601795	15.72944	22.34319
33	8.784514	28.58152374	1.204512	38.48027
34	0.894744	11.51132581	0.662238	15.56174
35	10.14732	41.456112	11.79059	31.09153
36	0.291324	4.2974377	0.169157	5.156138
37	17.13341	64.41551829	21.98242	67.45217
38	6.925581	29.751336	7.875085	31.00645
39	4.857157	38.19658808	6.135127	36.71597
40	6.912462	49.42179236	5.516562	55.57521
41	10.03727	34.65609291	13.71881	37.56976
42	16.98298	28.67110678	13.11093	24.5721
43	9.673159	29.76651275	11.90128	33.57092
44	18.0645	38.75305505	5.997651	43.59561
45	10.45172	29.27614836	8.261964	32.40166
46	0.891537	15.29851164	1.860124	19.73824
47	2.105975	25.72642754	5.27501	31.47027
48	12.72664	59.772115	13.66063	55.73892
49	4.697945	38.002715	6.308153	37.79076
50	8.159302	29.708174	9.097149	32.88905

**Table 6:** SP-A and SP-D level in BALF of each group.

Groups	SP-A (ng/ml)	SP-D (ng/ml)
Con	473.9 ± 60.41	44.50 ± 16.78
COPD	983.67 ± 84.05^∗^	376.7 ± 58.08
COPD + anti-miR-149-3p	1058.10 ± 98.64^∗^	396.7 ± 94.12
*F*	147.818	93.825
*P*	<0.001	<0.001

^∗^ *P* < 0.05, compared with Con group.

## Data Availability

The data used to support the findings of this study are included within the article.
